# *Lactobacillus plantarum* and *Lactobacillus brevis* Alleviate Intestinal Inflammation and Microbial Disorder Induced by ETEC in a Murine Model

**DOI:** 10.1155/2021/6867962

**Published:** 2021-09-21

**Authors:** Xuebing Han, Sujuan Ding, Yong Ma, Jun Fang, Hongmei Jiang, Yi Li, Gang Liu

**Affiliations:** College of Bioscience and Biotechnology, Hunan Agricultural University, Hunan Provincial Engineering Research Center of Applied Microbial Resources Development for Livestock and Poultry, Changsha, Hunan 410125, China

## Abstract

The purpose of this research is to explore the positive effects of *Lactobacillus plantarum* and *Lactobacillus brevis* on the tissue damage and microbial community in mice challenged by Enterotoxigenic *Escherichia coli* (ETEC). Twenty-four mice were divided into four groups randomly: the CON group, ETEC group, LP-ETEC group and LB-ETEC group. Our results demonstrated that, compared with the ETEC group, the LP-ETEC and LB-ETEC groups experienced less weight loss and morphological damage of the jejunum. We measured proinflammatory factors of colonic tissue and found that *L. plantarum* and *L. brevis* inhibited the expression of proinflammatory factors such as IL-*β*, TNF-*α*, and IL-6 and promoted that of the tight junction protein such as claudin-1, occludin, and ZO-1. Additionally, *L. plantarum* and *L. brevis* altered the impact of ETEC on the intestinal microbial community of mice, significantly increased the abundance of probiotics such as *Lactobacillus*, and reduced that of pathogenic bacteria such as *Proteobacteria*, *Clostridia*, *Epsilonproteobacteria*, and *Helicobacter*. Therefore, we believe that *L. plantarum* and *L. brevis* can stabilize the intestinal microbiota and inhibit the growth of pathogenic bacteria, thus protecting mice from the gut inflammation induced by ETEC.

## 1. Introduction

Diarrhea induced by Enterotoxigenic *Escherichia coli* (ETEC) is a major challenge to newborn piglets and weaned piglets. The condition increases occurrences of morbidity and mortality, which results in huge economic losses in the global swine industry [1]. ETEC can colonize in the small intestine, increase the expression of proinflammatory factors, cause intestinal barrier damage, and eventually lead to the development of intestinal inflammation [2–4]. Previous studies have proven that probiotics are vital to prevent and treat cancer [5], inflammatory bowel disease (IBD) [6], irritable bowel syndrome [7], and other diseases. In addition, probiotics can counteract the inflammation caused by ETEC [8, 9]. Most notably, probiotics are considered the only effective feed additives that protect against pathogens. In view of the injury and economic losses caused by intestinal inflammation such as diarrhea, colorectal cancer, and IBD, it is necessary to study probiotics, which are used as feed additives to inhibit pathogens and promote intestinal health.

Lactic acid bacteria, a kind of probiotic that exists in human and animal intestines, can enhance the immune system by inhibiting the expression of proinflammatory cytokines or promoting that of anti-inflammatory cytokines [10, 11]. *Lactobacillus plantarum* is a major species of lactic acid bacteria, with a variety of probiotic characteristics, gastrointestinal transport tolerance, and anti-inflammatory properties [12]. Previous studies have shown that the supplementation of *L. plantarum* in the diet can promote the gastrointestinal health of weaned piglets [13] and improve the antioxidant status and growth performance of piglets [14]. It is worth noting that studies on the effect of *L. plantarum* on various cancers are also being actively carried out [15, 16]. As a close relative of *L. plantarum*, *L. brevis* also has a variety of probiotic characteristics, including tolerance to acid and bile, adhesion to intestinal cells, and the ability to survive through the gastrointestinal tract [17, 18]. These characteristics of *L. brevis* enable it to maintain the homeostasis of the intestine, improve the barrier function of intestinal epithelium under oxidative stress, and reduce intestinal inflammation in the mouse model [19, 20].

In this study, ETEC was used to induce intestinal inflammation in a mouse model. And we explored the protective effects of *L. plantarum* and *L. brevis* on the injury induced by ETEC in the murine model, observed the changes in the amounts of cytokines and tight junction proteins, and determined the effects of these two probiotics on intestinal flora.

## 2. Materials and Methods

### 2.1. Bacteria

The strains *Lactobacillus plantarum* GL17, *Lactobacillus brevis* AY858, and Enterotoxigenic *Escherichia coli* used in this study were stored in Hunan Agricultural University (Changsha, China). *L. plantarum* GL17 and *L. brevis* AY858 were cultured in MRS broth at 37°C for 24 hours, while ETEC was cultured in a Luria-Bertani liquid medium at 37°C for the same time. To quantify the colonies, the cultured bacterial fluid was transferred to the corresponding solid medium and incubated at 37°C for 24 hours. Then, the bacterial samples of both probiotics and ETEC were centrifuged and suspended in sterile normal saline solution at 5 × 10^10^ CFU/mL and 1 × 10^8^ CFU/mL, respectively.

### 2.2. Animals and Experimental Design

**Figure 1:**
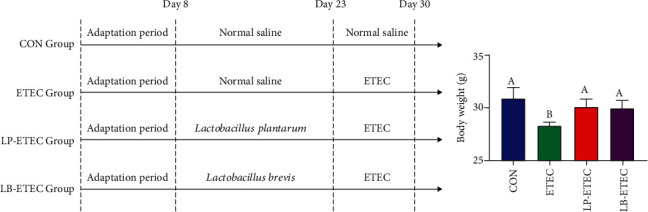
Impact of *Lactobacillus plantarum* and *Lactobacillus brevis* on body weight. The experimental process (a) and body weight (b). Data are mean ± SD (*n* = 6). Without a common letter mark indicates significant differences (*P* < 0.05).

The experiment was approved by the Animal Care and Use Committee of Hunan Agricultural University. Twenty-four eight-week-old ICR mice from the Shanghai Laboratory Animal Central (Changsha, China) were housed in a pathogen-free environment for seven days of adaptation. Then, the mice were divided into four groups randomly: the control group (CON), the ETEC group (ETEC), the *L. plantarum* GL17 treatment group (LP-ETEC), and the *L. brevis* AY858 treatment group (LB-ETEC). There were six mice in each group, and the basic diet and water for all mice were not limited. The experiment lasted for 21 days (Figure 1(a)). During the first 14 days of the experiment, the LP-ETEC group and the LB-ETEC group were given *L. plantarum* GL17 and *L. brevis* AY858, respectively, by gavage every day, while the control group and the ETEC group were given sterile saline every day. From the 15th day of the experiment, the ETEC group, LP-ETEC group, and LB-ETEC group were given ETEC by gavage until the end of the experiment. On the 21st day, all mice were killed, and a part of the jejunum was fixed in paraformaldehyde for histomorphological analysis, while the contents of the colon were collected and frozen in liquid nitrogen for the determination of microbial diversity.

### 2.3. Jejunum Histopathology

The jejunum samples fixed in the paraformaldehyde were dehydrated using ethanol gradient and embedded in paraffin. The samples were then stained with hematoxylin and eosin and observed under a microscope. The specific process is in accordance with methods used in previous research [21].

### 2.4. qPCR

The total RNA of frozen colonic tissue was extracted by TRIzol (Invitrogen, USA) according to the instruction. To determine the concentration of the extracted RNA, DNase I was used to treat RNA, and then, the concentration was determined by spectrophotometer at 260 nm. The primers used in the experiment are shown in Table 1, and the specific methods refer to a previous experiment [22]. Finally, the amplification reaction was carried out, and the calculation formula used to determine gene expression level refers to Ma et al. [23].

### 2.5. 16S Ribosomal RNA Amplicon Sequencing

To determine the diversity of microorganisms in the colonic contents, the microbial genomic DNA of colon contents was extracted by using the QIAamp DNA Stool Mini Kit firstly. Then, the concentration and purity of DNA were detected on 1% agarose gel, and the primers 357F (5′-ACTCCTACGGRAGGCAGCAG-3′) and 806R (5′-GGACTACHVGGGTWTCTAAT-3′) were used to amplify the V3-V4 region of 16S rDNA. After recovery and purification of PCR products, the library was prepared for sequencing analysis. Finally, the quality of off-machine data was controlled and optimized, and the composition of microbial communities was determined by OTU clustering. Moreover, the *α* diversity analysis was performed by mothur (Version 1.33.3).

### 2.6. Data Analysis

All of the data in the experiment are expressed as mean ± standard deviation (SD) and analyzed by one-way ANOVA and Tukey's multiple comparison test to compare the differences between the four groups (SPSS 21 software). *P* value < 0.05 was regarded as a significant difference.

## 3. Results

### 3.1. *L. plantarum* and *L. brevis* Inhibit the Weight Loss Induced by ETEC

The results of weight are shown in Figure 1(b). Compared with the CON group, ETEC reduced the weight of mice significantly (*P* < 0.05). When treated with *L. plantarum* GL17 and *L. brevis* AY858, the weight of mice increased significantly (*P* < 0.05). The results showed that these two probiotics reduced weight loss in the mice.

### 3.2. *L. plantarum* and *L. brevis* Inhibit the Development of Intestinal Injury Induced by ETEC

**Figure 2:**
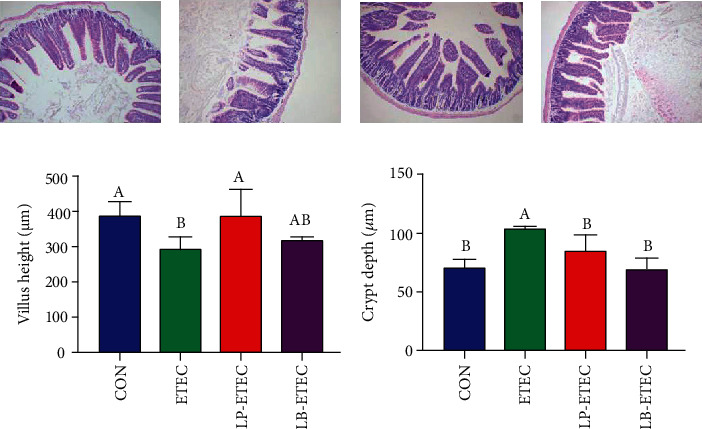
Impact of *Lactobacillus plantarum* and *Lactobacillus brevis* on jejunum tissue damage induced by ETEC. Images of jejunal tissue in the CON (a), ETEC (b), LP-ETEC (c), and LB-ETEC (d) groups; villus height (e); and crypt depth (f) in the four groups. Data are mean ± SD (*n* = 6). Without a common letter mark indicates significant differences (*P* < 0.05).

The results of histologic examination (Figure 2) showed that the height of jejunal villi decreased significantly (*P* < 0.05) and the crypt depth increased significantly (*P* < 0.05) after ETEC attack. Compared with the ETEC group, the mice in the LP-ETEC group had increased villus heights and reduced crypt depth significantly (*P* < 0.05). Similar results were observed in the LB-ETEC group, with a slight increase in villus heights and a significant decrease in crypt depth (*P* < 0.05). These results indicated that both of those two probiotics can reduce the intestinal damage caused by ETEC, especially *L. plantarum* GL17, which made the LP-ETEC group return to the same level as the CON group.

### 3.3. *L. plantarum* and *L. brevis* Affect Inflammation and Intestinal Barrier

**Figure 3:**
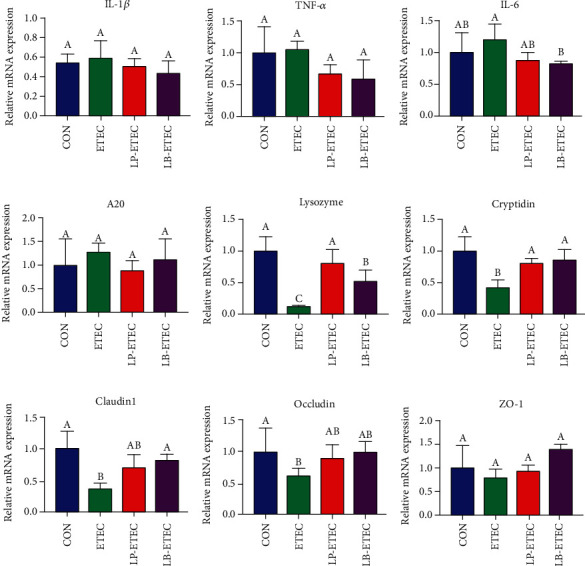
Impact of *Lactobacillus plantarum* and *Lactobacillus brevis* treatment on the expressions of cytokines and tight junction protein. 1The relative gene expression level determined by qPCR of (a) IL-1*β*, (b) TNF-*α*, (c) IL-6, (d) A20, (e) lysozyme, (f) cryptidin, (g) claudin-1, (h) occludin, and (i) ZO-1. Data are mean ± SD (*n* = 6). Without a common letter mark indicates significant differences (*P* < 0.05).

The expressions of cytokines in the colonic tissues were measured (Figures 3(a)–3(f)). Compared with the CON group, lysozyme and cryptidin in the ETEC group decreased significantly (*P* < 0.05), while no significant difference was observed in that of IL-1*β*, TNF-*α*, IL-6, and A20. When treated with *L. plantarum* GL17, lysozyme and cryptidin increased significantly (*P* < 0.05). Similarly, after the treatment of *L. brevis* AY858, a significant increase in lysozyme and cryptidin (*P* < 0.05) and a significant decrease in IL-6 (*P* < 0.05) were observed.

The expression of tight junction proteins is shown in Figures 3(g)–3(i). When attacked by ETEC, claudin-1 and occludin decreased significantly (*P* < 0.05), while ZO-1 remained almost unchanged. *L. plantarum* GL17 increased claudin-1 and occludin slightly, but there was no significant difference compared with the ETEC group. Similarly, no significant difference was observed in occludin between the LB-ETEC group and the ETEC group. However, compared with the ETEC group, claudin-1 in the LB-ETEC group increased significantly (*P* < 0.05).

### 3.4. *L. plantarum* and *L. brevis* Regulate Intestinal Microbes in Mice

**Figure 4:**
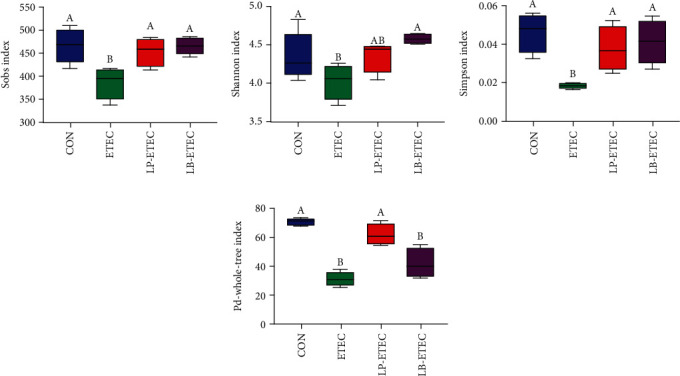
Impact of *Lactobacillus plantarum* and *Lactobacillus brevis* treatment on intestinal microbial diversity. (a) Sobs index; (b) Shannon index; (c) Simpson index; (d) PD-whole-tree index. Data are mean ± SD (*n* = 6). Without a common letter mark indicates significant differences (*P* < 0.05).

The results of intestinal microorganism diversity are shown in Figure 4. After the ETEC challenge, the Sobs index, Shannon index, Simpson index, and PD-whole-tree index decreased significantly (*P* < 0.05). After the treatment of *L. plantarum* GL17 and *L. brevis* AY858, these indexes increased and returned to normal level, especially the Sobs index and Simpson index, which were significantly different from those of the ETEC group (*P* < 0.05). Furthermore, the Shannon index in the LB-ETEC group and the PD-whole-tree index in the LP-ETEC group were significantly different from those in the ETEC group (*P* < 0.05). Therefore, both of these two probiotics have obvious protective effects on ETEC-induced decline of intestinal microbial diversity in mice.

**Figure 5:**
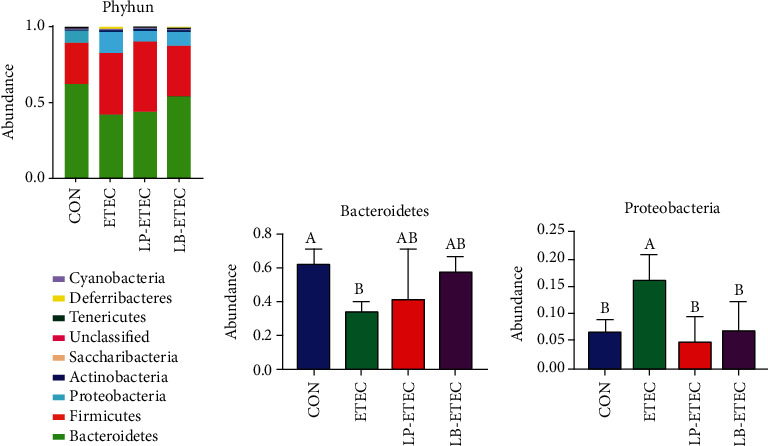
Impact of *Lactobacillus plantarum* and *Lactobacillus brevis* treatment on the microorganisms at the phylum level. (a) Relative abundance of microorganisms in the four groups at the phylum level; (b) abundance of *Bacteroidetes* in each of four groups; (c) abundance of *Proteobacteria* in each of four groups. Data are mean ± SD (*n* = 6). Without a common letter mark indicates significant differences (*P* < 0.05).

At the phylum level, there are nine kinds of microorganisms in each of four groups, of which *Bacteroidetes*, *Firmicutes*, and *Proteobacteria* accounted for more than 86% of all microorganisms (Figure 5(a)). The abundance of *Bacteroidetes* in the CON, ETEC, LP-ETEC, and LB-ETEC groups was 62.1485%, 42.3336%, 43.6535%, and 53.8593%, respectively. *Firmicutes* accounted for 26.6949%, 40.0591%, 46.6572%, and 33.6746%, respectively. The *Proteobacteria* abundance was 8.4628%, 14.4314%, 6.6691%, and 8.7466%, respectively. After being challenged by ETEC, *Bacteroidetes* in mice decreased significantly (*P* < 0.05). In contrast, the abundance of *Proteobacteria* showed the opposite trend, which was significantly higher than that of the CON group (*P* < 0.05) (Figures 5(b) and 5(c)). However, when treated with *L. plantarum* GL17 and *L. brevis* AY858, *Proteobacteria* in mice decreased significantly (*P* < 0.05).

**Figure 6:**
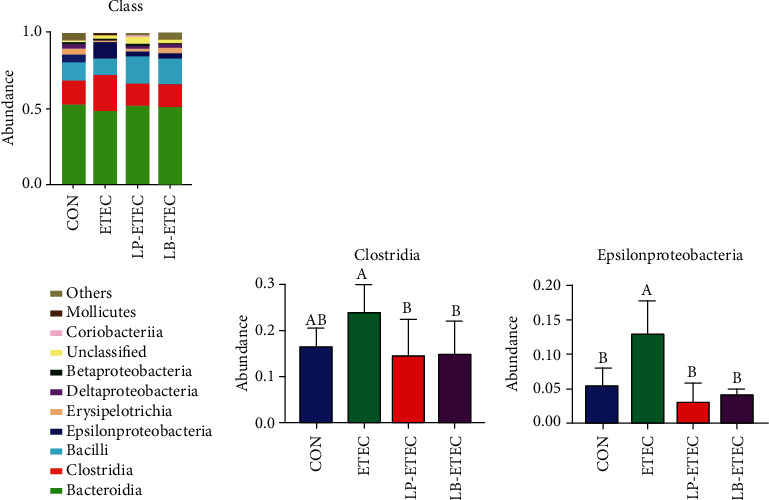
Impact of *Lactobacillus plantarum* and *Lactobacillus brevis* treatment on the microorganisms at the class level. (a) Relative abundance of microorganisms in the four groups at the class level; (b) abundance of *Clostridia* in each of four groups; (c) abundance of *Epsilonproteobacteria* in each of four groups. Data are mean ± SD (*n* = 6). Without a common letter mark indicates significant differences (*P* < 0.05).

The abundance of *Bacteroidia*, *Clostridia*, and *Bacilli* accounted for more than 80% of all microorganisms in the class level (Figure 6(a)). The abundance of *Bacteroidia* in the CON, ETEC, LP-ETEC, and LB-ETEC groups was 52.562%, 49.0004%, 52.9771%, and 51.7049%, respectively. *Clostridia* accounted for 16.7878%, 23.7975%, 14.2299%, and 14.7012%, respectively. The *Bacilli* abundance was 11.3105%, 10.0499%, 17.1775%, and 16.7497%, respectively. After being attacked by ETEC, the abundance of *Clostridia* and *Epsilonproteobacteria* increased, especially *Epsilonproteobacteria*, which increased significantly (*P* < 0.05) (Figures 6(b) and 6(c)). However, both *L. plantarum* GL17 and *L. brevis* AY858 significantly reduced the abundance of *Clostridia* and *Epsilonproteobacteria* (*P* < 0.05).

**Figure 7:**
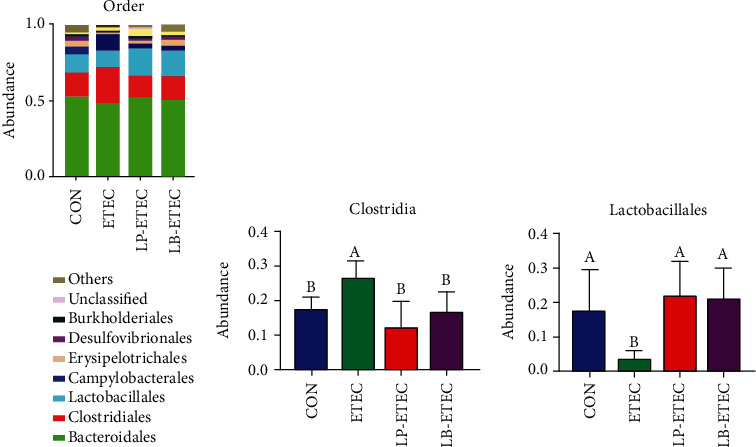
Impact of *Lactobacillus plantarum* and *Lactobacillus brevis* treatment on the microorganisms at the order level. (a) Relative abundance of microorganisms in the four groups at the order level; (b) abundance of *Clostridiales* in each of four groups; (c) abundance of *Lactobacillales* in each of four groups. Data are mean ± SD (*n* = 6). Without a common letter mark indicates significant differences (*P* < 0.05).

As for the order level, the abundance of *Bacteroidales*, *Clostridiales*, and *Lactobacillales* was the highest in the CON, LP-ETEC, and LB-ETEC groups (Figure 7(a)). The abundance of *Bacteroidales* in the CON, LP-ETEC, and LB-ETEC groups was 50.562%, 39.1805%, and 39.7049%, respectively. *Clostridiales* accounted for 17.1168%, 14.2299%, and 16.8516%, respectively. The abundance of *Lactobacillales* was 17.2176%, 22.0299%, and 21.248%, respectively. However, the top three most abundant microorganisms at the order level in ETEC group were *Bacteroidales*, *Clostridiales*, and *Campylobacterales*, accounting for 49.0004%, 25.221%, and 7.6303%, respectively. *Clostridiales* in the ETEC group increased significantly (*P* < 0.05) and *Lactobacillales* in the ETEC group decreased significantly (*P* < 0.05) compared with those in the CON group (Figures 7(b) and 7(c)). After the treatment of *L. plantarum* GL17 and *L. brevis* AY858, the abundance of *Clostridiales* and *Lactobacillales* had changed significantly (*P* < 0.05).

**Figure 8:**
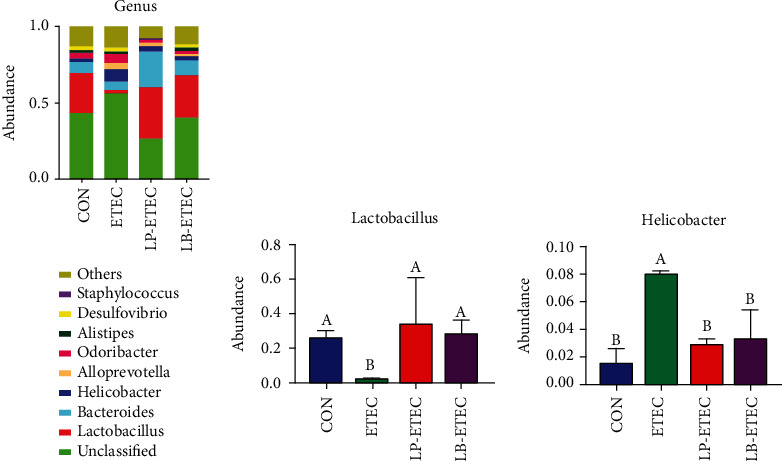
Impact of *Lactobacillus plantarum* and *Lactobacillus brevis* treatment on the microorganisms at the genus level. (a) Relative abundance of microorganism in the four groups at the genus level; (b) abundance of *Lactobacillus* in each of four groups; (c) abundance of *Helicobacter* in each of four groups. Data are mean ± SD (*n* = 6). Without a common letter mark indicates significant differences (*P* < 0.05).

Eight representative microbial genera in the four groups were selected and analyzed (Figure 8(a)). In the genus of microorganisms that have been classified, *Lactobacillus*, *Bacteroides*, and *Helicobacter* were the three main microorganisms among the groups of CON, LP-ETEC, and LB-ETEC. The abundance of *Lactobacillus* in the CON, LP-ETEC, and LB-ETEC groups was 26.08%, 33.5332%, and 27.7802%, respectively. *Bacteroides* accounted for 7.4573%, 23.1571%, and 9.449%, respectively. The abundance of *Helicobacter* was 1.486%, 2.9112%, and 3.2437%, respectively. However, the top three most abundant microbial genera in the ETEC group were *Bacteroides*, *Helicobacter*, and *Alloprevotella*, accounting for 5.9428%, 8.0295%, and 4.6711%, respectively. After being challenged by ETEC, the abundance of *Lactobacillus* reduced significantly (*P* < 0.05), while that of *Helicobacter* increased significantly (*P* < 0.05) (Figures 8(b) and 8(c)). However, when treated with *L. plantarum* GL17 and *L. brevis* AY858, the abundance of *Lactobacillus* and *Helicobacter* changed significantly (*P* < 0.05) and returned to the normal level.

## 4. Discussion

The protective effects of *L. plantarum* GL17 and *L. brevis* AY858 on the injury induced by ETEC were explored in this study. The results showed that both probiotics reduced the weight loss and morphological damage of the jejunum significantly. The expression of cytokines decreased, while that of the tight junction protein increased in mice treated with *L. plantarum* GL17 and *L. brevis* AY858. Meanwhile, *L. plantarum* GL17 and *L. brevis* AY858 restored the colonic microbial diversity to the normal level in mice challenged by ETEC and increased the relative abundance of *Lactobacillus*. In contrast, *L. plantarum* GL17 and *L. brevis* AY858 reduced the relative abundance of *Proteobacteria*, *Clostridia*, *Epsilonproteobacteria*, and *Helicobacter* in the colon after ETEC challenge.

The surface of intestinal mucosa, which is the largest surface of the human body, contacts with the external environment continuously [24]. Columnar epithelial cells are arranged into the intestinal epithelium and folded into crypts or concave [25]. These fully differentiated epithelial cells protect the body from potentially harmful microorganisms and viruses in the intestinal microenvironment [26]. Intestinal villi are critical components of the intestine that can increase the absorption area and promote the absorption of nutrients [27]. Endotoxin produced by ETEC can cause a variety of morphological changes of the intestinal tract, such as the increase of mucosal crypt depth, decrease of villus height, and submucosal edema [28]. Probiotics can reduce the morphological damage caused by the endotoxin, which can increase the height of villi and promote the growth of piglets [29, 30]. A previous study has shown that piglets fed with *L. plantarum* displayed higher villus height and lower crypt depth in the jejunum [1]. The same results were obtained in this experiment. *L. plantarum* and *L. brevis* protected the structural integrity of the jejunum, as well as the ability of absorbing nutrients. However, the protective effect of these two probiotics on villi is slightly different; *L. plantarum* is better than *L. brevis* at restoring the height of intestinal villi.

ETEC produces heat-labile enterotoxin (LT), and the LTA subunit of it, together with ADP-ribosylation factor, can induce the ribosylation of Gs*α* [31]. At that time, the adenylate cyclase of the target cell is uncontrolled, converting ATP to cAMP continuously. The increase of cAMP will not only activate the NF-*κ*B signaling pathway and produce a large number of inflammatory factors [32] but also activate the MAPK signaling pathway, resulting in the dislocation of tight junction proteins and impairment of intestinal barrier function [33]. The increase of proinflammatory factors, including IL-1*β*, TNF-*α*, and IL-6, will aggravate intestinal inflammation and promote the occurrence of colorectal cancer. In this experiment, although the difference was insignificant, the proinflammatory factors showed an upward trend after ETEC challenge, which indicated that ETEC increased intestinal inflammation in mice. Probiotics can reduce the inflammatory response by reducing the level of cytokines [12, 34, 35], which is also proven by our research. Although the difference was not significant, the proinflammatory factors in mice treated with *L. plantarum* and *L. brevis* showed a decreasing trend. Previous researches conducted *in vitro* have shown that ETEC can reduce the amounts of occludin in Small Intestinal Epithelial Cell Line- (IPEC-) 1 enterocytes of piglets [36], the permeability of tight junctions in IPEC-J2 enterocytes of piglets [37], and dislocation of ZO-1 Caco-2 cells of human [38]. However, probiotics increased the expression of tight junction proteins such as ZO-1, caudin-1, and occludin to protect cells [36, 39]. In this experiment, claudin-1 and occludin decreased significantly after ETEC challenge, while ZO-1 also showed a decreasing trend in mice, indicating that ETEC destroyed the intestinal barrier in mice. In contrast, *L. plantarum* and *L. brevis* increased the expression of the tight junction protein in mice challenged by ETEC. And the protective effect of *L. brevis* on the barrier function of mice was slightly higher than that of *L. plantarum*. Lysozyme is a critical bacteriostatic protein that strongly inhibits gram-positive bacteria [40], and cryptidin has a significant therapeutic effect on mice infected with *Salmonella Typhimurium* [41]. Therefore, the increase of these two substances in mice treated with *L. plantarum* and *L. brevis* may inhibit the growth of pathogenic bacteria and protect the health of mice.

There are lots of microbiota in the intestinal ecosystem, which play a significant role in host immunity and disease prevention. Therefore, unstable intestinal ecology may cause many diseases, such as ulcerative colitis and chronic diarrhea [42, 43]. ETEC reduced the intestinal microbial diversity of mice, while *L. plantarum* and *L. brevis* restored it to the normal level. The PD-whole-tree index of mice treated with *L. plantarum* was significantly higher than that of mice only challenged by ETEC, but there was no significant difference in the Shannon index. However, the results of mice treated with *L. brevis* showed the opposite results. This indicates that these two probiotics have almost the same effect on the recovery of intestinal microbial diversity in mice.

*Proteobacteria* is known to be the most disease-related intestinal microorganism, causing metabolic diseases and intestinal inflammation [44–46]. In this phylum, most microorganisms are human pathogens. Our study found that both *L. plantarum* and *L. brevis* inhibited the increase of *Proteobacteria* caused by ETEC significantly and made it return to the normal level, thus reducing the risk of intestinal inflammation in mice. Both *Clostridia* and *Epsilonproteobacteria* are pernicious bacteria that can cause digestive tract diseases in children. Tissue infection and intestinal diseases are often caused when those two bacteria enter the body of humans and other animals [47, 48]. *Clostridia* is involved in the development of necrotizing enterocolitis, which is a digestive tract disease that can threaten the life of preterm neonates [47]. In this study, mice treated with *L. plantarum* and *L. brevis* decreased the abundance of *Clostridia* and *Epsilonproteobacteria*, as well as a lower risk of digestive tract disease. As we all know, *Lactobacillus* is a probiotic with the function of preventing infection, reducing incidences of diarrhea, and improving production performance [49]. Some microorganisms of this genus, such as *Lactobacillus rhamnosus* and *Lactobacillus reuteri*, can protect the tight junctional protein after infection and contribute to the gut barrier function [50, 51]. In this experiment, both *L. plantarum* and *L. brevis* increased the abundance of *Lactobacillus* significantly, which may contribute to the gut barrier function and protection against inflammation. *Helicobacter* is the most common source of infection in the world, and it is the main risk factor of gastric cancer. Due to the ability to adapt to extreme acidic environment, *Helicobacter* can establish persistent infection and relieve the regulatory function of the host, leading to the pathogenesis and cancer of the digestive tract [52]. *L. plantarum* and *L. brevis* inhibited the growth of such pathogenic bacteria significantly and reduced the risk of canceration in tissue. It can be seen from the results that there is no significant difference between *L. plantarum* and *L. brevis* in promoting the abundance of probiotics and inhibiting that of pathogenic bacteria, indicating that they have almost the same effects on inhibiting intestinal inflammation and canceration.

## 5. Conclusion

The results in this experiment showed that *L. plantarum* and *L. brevis* can prevent the weight loss and intestinal injury caused by ETEC effectively, reduce the production of inflammatory factors, and strengthen the intestinal barrier function. Moreover, both of these two probiotics can stabilize the microbial community structure of intestine in mice, increase the abundance of probiotics such as *Lactobacillus*, and reduce the abundance of pathogenic bacteria such as *Proteobacteria*, *Clostridia*, *Epsilonproteobacteria*, and *Helicobacter*. Therefore, *L. plantarum* and *L. brevis* showed similarly effective inhibition on intestinal injury induced by ETEC and the ability to improve immune function. In summary, the feasibility and effectiveness of *L. plantarum* and *L. brevis* in the treatment of intestinal inflammation are demonstrated in our study, which provides a basis for further study of these two probiotics and their impact on intestinal inflammation such as diarrhea and colon cancer.

## Figures and Tables

**Table 1:** The primers for this study.

Primer	Name	5′→3′ sequence
IL-1*β*	IL-1*β*_F	ATGAAAGACGGCACACCCAC
	IL-1*β*_R	GCTTGTGCTCTGCTTGTGAG

TNF-*α*	TNF-*α*_F	ACCCTGGTATGAGCCCATATAC
	TNF-*α*_R	ACACCCATTCCCTTCACAGAG

IL-6	IL-6_F	GAGGATACCACTCCCAACAGACC
	IL-6_R	AAGTGCATCATCGTTGTTCATACA

Claudin-1	Claudin-1_F	GGGGACAACATCGTGACCG
	Claudin-1_R	AGGAGTCGAAGACTTTGCACT

Occludin	Occludin_F	TTGAAAGTCCACCTCCTTACAGA
	Occludin_R	CCGGATAAAAAGAGTACGCTGG

ZO-1	ZO-1_F	GATCCCTGTAAGTCACCCAGA
	ZO-1_R	CTCCCTGCTTGCACTCCTATC

Lysozyme	Lysozyme_F	GCCAAGGTCTAACAATCGTTGTGAGTTG
	Lysozyme_R	CAGTCAGCCAGCTTGACACCACG

Cryptidin	Cryptidin_F	TCAAGAGGCTGCAAAGGAAGAGAAC
	Cryptidin_R	TGGTCTCCATGTTCAGCGACAGC

A20	A20_F	AAACCAATGGTGATGGAAACTG
	A20_R	GTTGTCCCATTCGTCATTCC

## Data Availability

The data of this study is available from the correspondence authors upon reasonable request.
